# Exciton Condensation in Molecular-Scale van der Waals Stacks

**DOI:** 10.1021/acs.jpclett.1c02368

**Published:** 2021-10-06

**Authors:** Anna O. Schouten, LeeAnn M. Sager, David A. Mazziotti

**Affiliations:** Department of Chemistry and The James Franck Institute, The University of Chicago, Chicago, Illinois 60637, United States

## Abstract

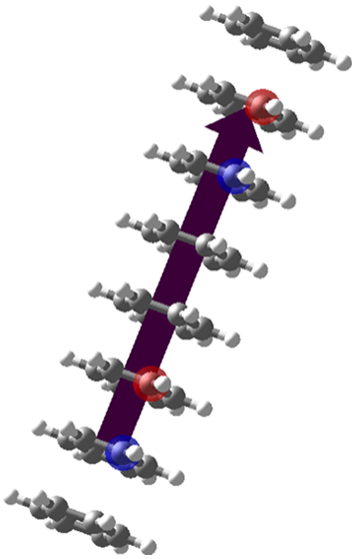

Recent experiments have realized the Bose–Einstein condensation of excitons, known as exciton condensation, in extended systems such as bilayer graphene and van der Waals heterostructures. Here we computationally demonstrate the beginnings of exciton condensation in multilayer, molecular-scale van der Waals stacks composed of benzene subunits. The populations of excitons, which are computed from the largest eigenvalue of the particle-hole reduced density matrix (RDM) through advanced variational RDM calculations, are shown to increase with the length of the stack. The large eigenvalue indicates a nonclassical long-range ordering of the excitons that can support the frictionless flow of energy. Moreover, we use chemical substitutions and geometric modifications to tune the extent of the condensation. Results suggest exciton condensation in a potentially large family of molecular systems with applications to energy-efficient transport.

Excitons—bound pairs of electrons and holes—can undergo a Bose–Einstein-like condensation into a single quantum state. The consequent superfluidity^1−4^ in such excitonic condensates enables frictionless flow of electrons and holes, presenting the possibility for dissipationless transfer of energy.^5,6^ Because of the fleeting excitonic lifetime,^7^ experimental observation of exciton condensation has proven difficult without either the coupling of excitons to photons, forming polaritons,^8,9^ or the use of prohibitively high magnetic fields and/or prohibitively low temperatures.^9−14^ Much contemporary experimental and theoretical investigation has centered on exciton condensation in bilayer systems^15−28^ as the two layers in such systems form a quantum well, which allows electrons in one layer to become bound to holes in the other layer to form a relatively long-lived, delocalized exciton,^5,7^ ideal for condensation applications. Indeed, in recent literature, exciton condensation has been observed—computationally and experimentally—in bilayer structures^22,24^ as well as multilayer van der Waals heterostructures.^19,25,27^ Moreover, recent theoretical results by Safaei and Mazziotti^24^ show that exciton condensation can potentially occur in bilayers of nonextended molecules. For multilayer systems, the excitons can flow through the stack to transfer energy. Collectively, these results suggest exploring exciton condensation in multilayer, molecular-scale systems with an investigation into the factors influencing the presence and extent of such condensation.

To that end, we analyze exciton condensation in multilayer, molecular-scale van der Waals stacks composed of two to six benzene and substituted-benzene subunits. Stacked benzene and benzene derivatives have π–π interactions which allow for transport between layers similar to a semiconductor, making them useful for applications in electron transport and conduction.^29−31^ These same characteristics can be beneficial in allowing exciton formation and condensation. Exciton condensation is characterized using a computational signature; a large eigenvalue (λ_*G*_ > 1) in the modified particle–hole reduced-density matrix (RDM)^24,32^ indicates the number of condensed excitons (i.e., excitons in a single particle-hole function). Using this signature, we demonstrate an increase in the degree of exciton condensation with the number of benzene subunits in the stack (system size). Additionally, the angle of rotation (θ) between each benzene layer in the bilayer system is varied from 0° to 60°, establishing that the greatest character of exciton condensation (the largest λ_*G*_) occurs for systems composed of aligned benzene subunits (θ = 0°, 60°, 120°, ...). Finally, substituent effects are probed by introducing electron-donating and electron-withdrawing substituents to each of the benzene subunits for all stacks. Both electron donation from the – NH_2_ substituent as well as the electron withdrawal from the – F substituent suppress the degree of exciton condensation, although the increase in condensation with the number of layers is still observed for the stacks composed of substituted-benzene subunits. Computations are performed with an advanced electronic structure method, the variational two-electron RDM (2-RDM) method,^33−48^ that has been shown to recover the strong electron correlation required to describe entanglement-driven phenomena in organometallic chemistry such as ligand noninnocence,^47^ nonsuperexchange mechanisms, and spin crossovers. The demonstration of potential exciton condensation in the van der Waals benzene stacks and the concomitant exploration of substituent and geometric effects will aid in the effort to rationally design molecular-scale exciton condensates that can be experimentally explored for possible utility in efficient energy transport.

Vertical stacks of benzene molecules, ranging from two to six layers, are used to examine exciton condensation in multilayer, molecular systems. Exciton condensation appears for an interlayer spacing range from about 1.75 to 3 Å (see the Supporting Information for more details). Keeping with prior investigation,^24^ interlayer distances are set to 2.5 Å for all calculations in this study. To compute the eigenvalues that act as computational signatures of exciton condensation,^24,32^ the particle–hole reduced-density matrix (RDM) is calculated for each molecular system (see Methods). The calculations describe excitons as collective excitations that connect the ground and excited electronic states. In this case, the excitons are in the triplet spin state. Note that a large eigenvalue for the particle–hole RDM (λ_*G*_ > 1) is evidence of the presence of exciton condensation with the magnitude of the eigenvalue indicating the extent of condensation. Calculations are reported below for two basis sets—sto-6g and cc-pvdz—to demonstrate qualitative consistency between basis sets; additional investigation of basis set dependence is described in the Supporting Information.

The signature of exciton condensation versus the number of layers in the stack is shown in Figure 1. From the sto-6g calculated eigenvalues, we note an almost-linear increase in condensate character with an increase in the number of layers in the stack. In fact, the signature of condensation can be fitted to
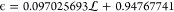
1where ϵ represents the eigenvalue and  represents the number of layers with an *R*^2^ of 0.9914. This fit allows us to extrapolate with reasonable accuracy the extent of exciton condensation in larger benzene stacks without the expense associated with direct calculation of the signature of condensation. The cc-pvdz calculated eigenvalues are consistent with the pattern of increasing condensate character with the number of layers, but show some deviations from linearity, seen by the fit
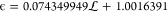
2with an *R*^2^ of 0.9653. Results from both basis sets demonstrate an increase in exciton condensation with the number of layers in the stack. To visualize the excitonic systems probed, we compute the probability of the hole as a function of the particle localized in a specified atomic orbital—for a given exciton (see Methods for more information). Specifically, Figure 2 illustrates the “exciton density” for the condensed excitonic mode in the six-layer stack, with the red atomic orbital representing the constrained location of the electron and with the purple and green representing the hole density (and the relative phases of its amplitude) corresponding to the specified electron. This image demonstrates that for the excitonic mode exhibiting condensation, hole density is delocalized through the entire stack with the greatest density on layers adjacent to the electron’s layer and with minimal density in the same layer as the electron. The density of the particle for a localized hole is identical to the density of the hole for a localized particle (see the Supporting Information for more details). The “exciton density” for each stack demonstrates similar exciton delocalization with both basis sets.

**Figure 1:**
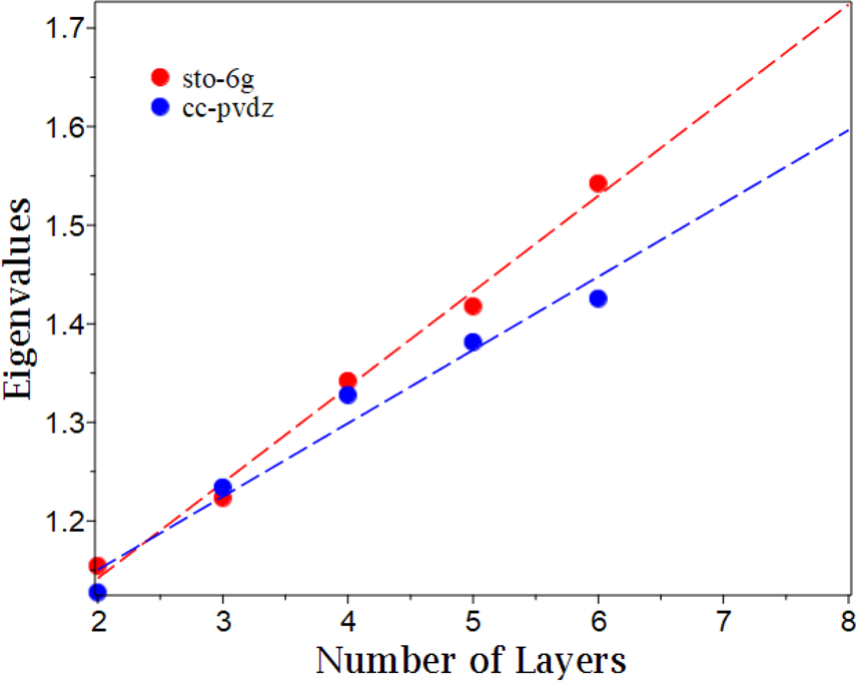
Plot of the number of layers versus the eigenvalues of the particle–hole RDM for the multilayer benzene for basis sets sto-6g and cc-pvdz. Dotted lines denote a linear fit of the data.

**Figure 2:**
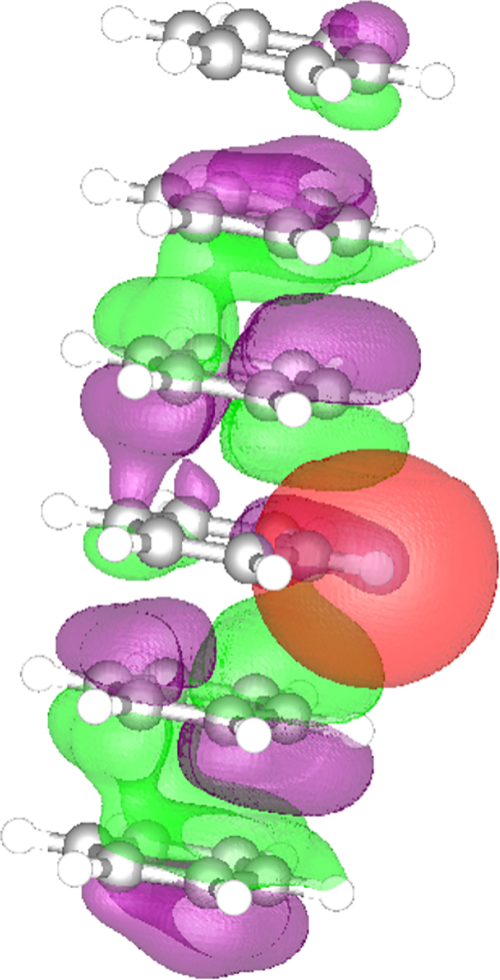
Visualization of “exciton density” for the six-layer system, with the red atomic orbital representing the constrained location of the electron and with the purple and green representing the hole density corresponding to the specified electron.

To assess the influence of interlayer angle on the degree of exciton condensation, we present the signature of exciton condensation for two-layer stacks with one benzene molecule rotated by θ degrees from θ = 0°, 10°, 20°, 30°, 40°, 50°, 60° relative to the other benzene. Because the previous results show qualitative consistency between basis sets, only the sto-6g basis set was used for these and subsequent calculations. Figure 3a depicts this internal rotation, where initially, for θ = 0°, the benzene molecules are aligned, and the rotation moves them out of alignment by θ degrees. As can be seen in Figure 3b, the degree of exciton condensation follows a sinusoidal pattern with the maximum occurring when the benzene layers are completely aligned (at 0° and 60°) and the minimum occurring when the layers are antialigned (30°). Because of the *D*_6*h*_ symmetry of benzene, this pattern is expected to repeat with a period of 60°. Rotation of the central layer in the three-layer stack shows a similar pattern (see Supporting Information for details).

**Figure 3:**
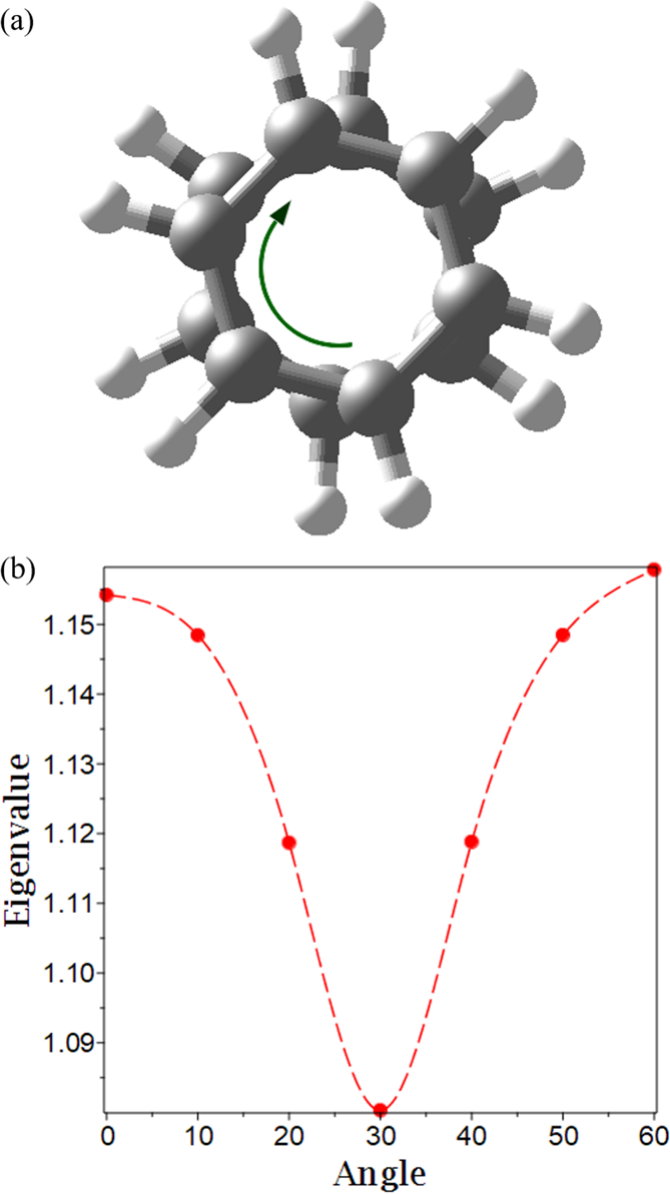
(a) Rotation of layers relative to one another. (b) Plot of the eigenvalues versus the angle. The interpolation is to demonstrate that intermediate angles are possible.

Overall, this trend indicates that exciton condensation of the benzene stack is maximized when the benzene molecules are aligned. Nevertheless, the condensate is relatively stable to angular deviations about the optimal alignment.

The effect of electron donating and electron withdrawing groups on exciton condensation is explored by probing the signature of exciton condensation for stacks composed of the substituted-benzene molecules aniline and fluorobenzene, the bilayer systems of which are shown in Figure 4a,b. Note these substituents are aligned for simplicity as our initial investigation of bilayer systems demonstrated that the relative positions (aligned, ortho, meta, para) of the substituents in different layers relative to each other has little impact on exciton condensation (see the Supporting Information for more details).

**Figure 4:**
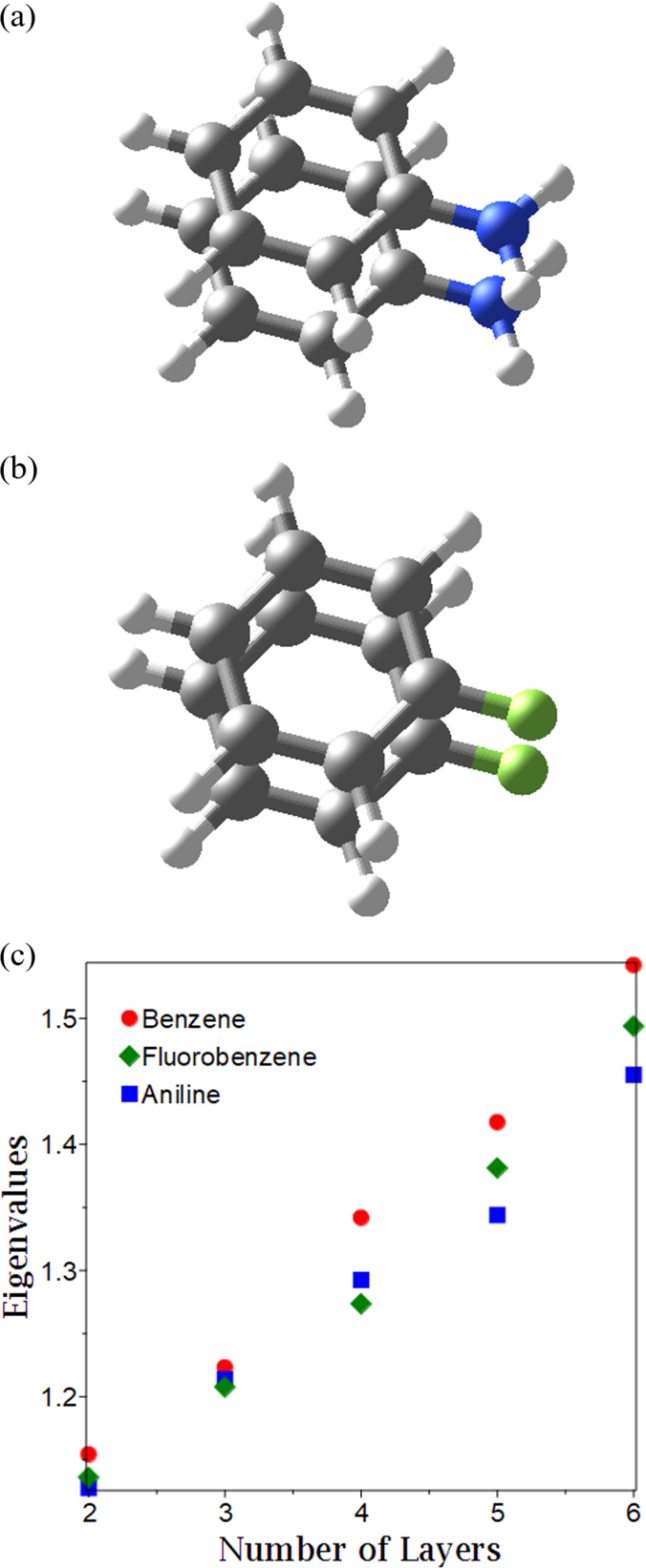
(a) Molecular picture of the aniline double-layer. (b) Molecular picture of the fluorobenzene double-layer. (c) Comparative plot of the growth of the signature of exciton condensation with the number of layers for benzene, aniline, and fluorobenzene.

As with benzene, the large eigenvalue increases with the number of layers for both aniline and fluorobenzene. However, as shown in Figure 4, relative to benzene, the large eigenvalues in both aniline and fluorobenzene are lower in magnitude. The decrease in eigenvalues indicates that the presence of either electron withdrawing or donating groups reduces exciton condensation in the systems probed. As both electron withdrawing and donating groups create an imbalance of electrons and holes, the addition of these substituents to each layer likely reduces the number of electron–hole pairs able to form excitons and hence participate in a condensate.

To investigate this further, we conducted a calculation of a bilayer structure with an amine subunit in one layer and a fluorine subunit in the other. As having one electron donating subunit and one electron withdrawing subunit creates a greater imbalance of electrons and holes, the degree exciton condensation is expected to decrease as compared to when the subunits are of matching type. Indeed, we observe that the signature of exciton condensation decreases to a smaller value (λ_*G*_ = 1.1241) than for a bilayer with either no substituents or the same substituent in both layers. It is significant to note, however, that in all cases there is still evidence of exciton condensation, as seen by the large eigenvalues. This is consistent with the experimental results of Kim et al.^22^ that demonstrate exciton condensation still occurs in graphene bilayers with electron–hole imbalances up to 30%.

In this study, we probe the presence and extent of exciton condensation in multilayer stacks of benzene molecules and demonstrate that the extent of condensation increases with the number of layers in the stack. Indeed, even in the presence of electron donating or withdrawing groups, this trend holds. Additionally, consistent with previous experimental results,^22^ we show that an imbalance of electrons and holes, in this case caused by the presence of electron donating or withdrawing groups, reduces but does not eliminate exciton condensation. Finally, we demonstrate that exciton condensation in benzene stacks is not impervious to geometric disruption; when one benzene layer in a bilayer is rotated relative to the other, the extent of exciton condensation is reduced as a function of the rotation angle. Overall, maximum exciton condensation is observed for aligned, nonsubstituted benzene molecules in stacks with the largest number of layers probed. While these methods do not provide a prediction of the expected condensation temperature or excitonic lifetime, the proximity of the layers would be expected to reduce the distance between the electron and hole and thus the effective radius of the exciton, resulting in a relatively large binding energy (>0.1 eV).^18^ A large binding energy would suggest a long excitonic lifetime and high condensation temperature. The predicted condensation could be experimentally verified by observing superfluidity via counterflow transport;^7^ recent studies have also used observation of electroluminescence^27^ and momentum-resolved electron energy-loss spectroscopy^21^ to probe condensation.

Exploration of the degree of exciton condensation in molecular-scale van der Waals stacks composed of benzene subunits provides evidence for a potentially large family of molecular-scale exciton condensates. While such condensates have been recently realized in extended systems such as graphene bilayers and van der Waals heterostructures, realizing exciton condensation in nonextended molecules would open new opportunities for constructing and tuning condensates synthetically. The present study reveals that the same factors that contribute to exciton condensation in extended systems—such as the use of bilayers or multilayers to facilitate particle–hole pairing—are equally important in molecules. Moreover, we show that substituents and orientations can be chemically tuned for optimal condensation. Like their extended analogues, molecular-scale exciton condensations exhibit nonclassical long-range order that supports the dissipationless flow of energy with potential applications in the design of more energy-efficient molecular structure and devices.

## Methods

Molecular geometries of benzene, aniline, and fluorobenzene were obtained from the PubChem database.^49−51^ The 2-RDM was variationally calculated directly from the molecular structures of the stacks.^33−48^ The energy for the molecule was optimized as a function of the 2-RDM, constrained by the general conditions for a density matrix as well as the 2-positive (DQG) *N*-representability conditions.^37,44,48,49,52^ These conditions constrain the 2-RDM ^2^*D*, the two-hole RDM ^2^*Q*, and the particle-hole RDM ^2^*G* to each be positive semidefinite. The minimal Slater-type orbital (STO-6G) basis set was used for all computations. Active spaces were determined based on the number of π electrons in the stack, and increased with the number of layers in the stack. For example, stacks with two layers used active space [12,12] and stacks with three layers used active space [18,18]. Substituted stacks followed the same pattern for active space as stacks with no substituents.

The particle–hole RDM ^2^*G* is calculated from the 2-RDM ^2^*D* by linear mapping, given by the relation:
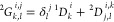
3where  is the 1-RDM calculated by contraction of the 2-RDM and  is the Kronecker delta. Because the particle–hole RDM always contains one large eigenvalue independent of exciton condensation that corresponds to ground-state-to-ground-state projection, the ground state resolution was removed producing a new particle–hole matrix:
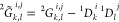
4

For , a large eigenvalue is indicative of exciton condensation.^24,32^ Eigenvalues, ϵ_*i*_, and eigenvectors, *v*_*i*_, were calculated from  using the following eigenvalue/eigenvector optimization:
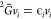
5

The exciton energy was calculated from the ^2^*G* matrix using the variational 2-RDM method.^35^ We use the implementation of the method in the Quantum Chemistry Package in Maple.^53,54^

As described above, the “exciton density” was visualized by constraining the location of the electron in a certain atomic orbital and calculating the resulting hole probability. A matrix of molecular orbitals in terms of atomic orbitals (*M*_MO,AO_) is calculated
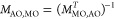
6A submatrix of the active orbitals, *M*_AO,MO_, is isolated from *M*_MO,AO_. The eigenvector of the  matrix corresponding to the large eigenvalue was reshaped as a matrix in the active molecular orbital basis, called *V*_max_. The matrices were multiplied as
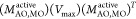
7to create a matrix representing specific electron atomic orbitals in terms of the coefficients of the contributions of the holes to other molecular orbitals.

## Supporting Information Available

Details of basis set exploration, layer spacing, rotation in the three-layer stack, density modeling, substituent position calculations, and molecular geometries (PDF)
