# Supplementary material for: Exciton Condensation in Molecular-Scale van der Waals Stacks
Source: J Phys Chem Lett. 2021 Oct 6;12(40):9906–11. doi: 10.1021/acs.jpclett.1c02368 (PMC8521519; doi:10.1021/acs.jpclett.1c02368)
Supplement: Supplementary file 1 — jz1c02368_si_001.pdf [file jz1c02368_si_001.pdf]

Supporting Information:
Exciton Condensation in Molecular-scale Van der Waals Stacks

Anna O. Schouten, Leann M. Sager, and David A. Mazziotti*
Department of Chemistry and The James Franck Institute, The University of Chicago, Chicago, IL 60637
(Dated: Submitted July 21, 2021; Revised August 19, 2021)

BASIS SETS

Number of layers	sto-6g	aug-cc-pvdz	cc-pvdz
**2**	1.1542	1.0000	1.1273
**3**	1.2232	1.2220	1.2338
**4**	1.3418	1.3311	1.3275
**5**	1.4175	1.3935	1.3812
**6**	1.5422	1.4387	1.4254

TABLE S1: Calculated eigenvalues of benzene stacks for sto-6g, aug-cc-pvdz, and cc-pvdz basis sets.

To probe the consistency of the results with different basis sets, we performed calculations with sto-6g, aug-cc-pvdz, and cc-pvdz. The calculated eigenvalues are shown in Table S1. As noted in the main text, the the sto-6g and cc-pvdz results are qualitatively consistent. The aug-cc-pvdz results differ only in the double-layer stack, which show no exciton condensation.

Basis Set	Eigenvalue
**sto-6g**	1.1542
**aug-cc-pvdz**	1.0000
**cc-pvdz**	1.1273
**6-31g***	1.1477
**dzp**	1.1271
**6-31+g***	1.1265
**6-31++g***	1.0000

TABLE S2: Calculated eigenvalues of the benzene double-layer for various basis sets.

Several basis sets were tested for the double-layer stack to investigate possible explanations for the difference in the double-layer stack with aug-cc-pvdz. As seen in Table S2, most basis sets gave qualitatively similar results that indicated exciton condensation in the double-layer. However, for augmented basis sets(aug-cc-pvdz and 6-31++g*) the eigenvalue shows no exciton condensation. This may be because the diffuse functions in the basis sets are sufficiently large to span the small double-layer system such that condensation no longer appears as long-range order, and is instead captured as short-range order. Supporting this, the eigenvalues for the triple-layer stack of both aug-cc-pvdz and 6-31++g*, 1.2220 and 1.2225, respectively, were similar to that of cc-pvdz, 1.2338, suggesting that as the size of the system increases exciton condensation again appears as long-range order.

SPACING

Figure S1 shows the eigenvalue as a function of layer spacing for the two-layer stack. There is a range from about 1.75 - 3 Å where exciton condensation is indicated by the eigenvalue. For the study a spacing of 2.5 Å is used, falling in this range.

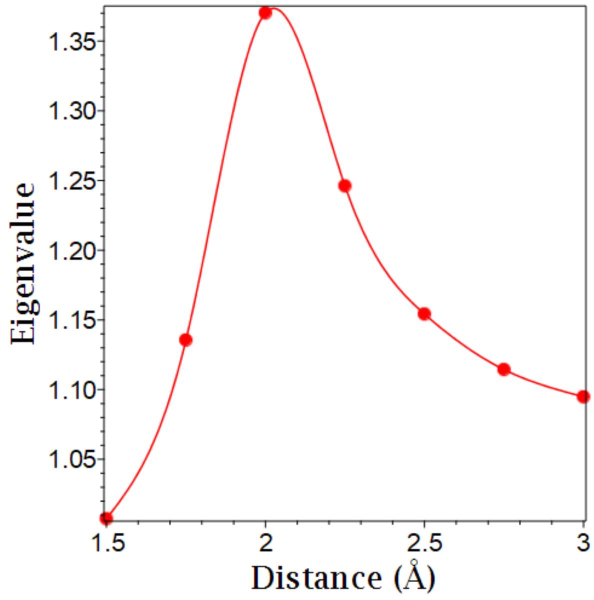

FIG. S1: Eigenvalue as a function of layer spacing to for two-layer benzene.

EXCITON DENSITY

The "exciton density" is modeled with the electron constrained and the hole probability plotted. In this case, the red represents the localized hole and the purple and green are the electron probability. The methods used for this calculation are described in detail in the Methods section. As seen in Figure S2, the density is identical to

* damazz@uchicago.edu

that modeled with a localized hole and electron probability.

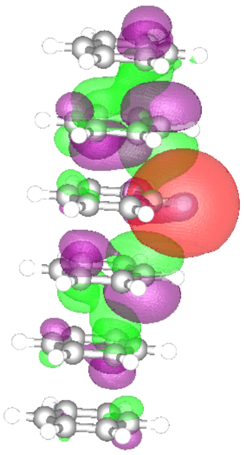

FIG. S2: Exciton density modeled with a constrained electron and predicted hole probability.

THREE-LAYER ROTATION

Rotation of the central layer in the three-layer stack is examined to determine how the additional layer would influence the change in the degree of condensation. The degree of condensation appears to be influence by the rotation of a central layer more than in the double layer, as seen in Figure S3, where the eigenvalue at 30° decreases by a larger amount than for the double-layer. However, the general pattern is the same as for rotation of a single layer in the double-layer stack.

SUBSTITUENT POSITIONS

For the substituted benzene sub-units aniline and fluorobenzene, the positions of the substituents relative to one another are probed for the double-layer stack. Table S3 shows the calculated eigenvalues for each position in both aniline and fluorobenzene double-layers, demonstrating the position had little influence on the degree of exciton condensation.

	Aligned	Ortho	Meta	Para
**Amine**	1.1042	1.0844	1.0926	1.0872
**Fluorine**	1.1368	1.1312	1.1379	1.1344

TABLE S3: Eigenvalues for double-layers with one substituent in each layer in the specified positions.

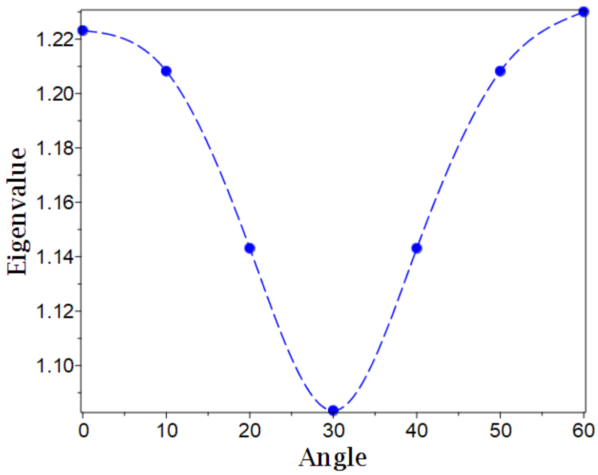

FIG. S3: Eigenvalues versus rotation angle for a three-layer stack with the center layer rotated.

COORDINATES

Coordinates were accessed from the PubChem database.

Benzene

12

H 2.13940 -1.25630 2.50010
H -0.01840 -2.48090 2.49990
C 1.20280 -0.70630 2.50000
C -0.01030 -1.39480 2.50000
C 1.21310 0.68840 2.50000
C -1.21310 -0.68840 2.50000
H 2.15770 1.22450 2.50000
H -2.15770 -1.22440 2.50000
C 0.01040 1.39480 2.49990
C -1.20280 0.70640 2.50010
H 0.01840 2.48080 2.50000
H -2.13930 1.25640 2.50010

Aniline

14

N -2.4046000000 0.0000000000 0.0005000000
C -0.9941000000 -0.0002000000 -0.0003000000
C -0.2969000000 1.2079000000 -0.0003000000
C -0.2965000000 -1.2080000000 -0.0003000000
C 1.0980000000 1.2080000000 0.0001000000
C 1.0984000000 -1.2078000000 0.0001000000
C 1.7957000000 0.0002000000 0.0003000000
H -0.8289000000 2.1558000000 -0.0003000000
H -0.8283000000 -2.1561000000 -0.0002000000
H 1.6411000000 2.1486000000 0.0002000000
H 1.6417000000 -2.1482000000 0.0002000000
H 2.8818000000 0.0004000000 0.0006000000
H -2.9109000000 -0.8755000000 -0.0005000000
H -2.9107000000 0.8756000000 -0.0006000000

Fluorobenzene
12

F -2.3436000000 0.0000000000 -0.0001000000
C -1.0043000000 0.0000000000 0.0002000000
C -0.3069000000 1.2080000000 0.0000000000
C -0.3068000000 -1.2079000000 0.0000000000
C 1.0880000000 1.2079000000 0.0000000000
C 1.0880000000 -1.2080000000 0.0000000000
C 1.7855000000 0.0000000000 0.0000000000
H -0.8501000000 2.1483000000 -0.0001000000
H -0.8501000000 -2.1483000000 -0.0001000000
H 1.6310000000 2.1485000000 -0.0001000000
H 1.6311000000 -2.1485000000 0.0000000000
H 2.8716000000 0.0001000000 0.0000000000
